# The effect of exposure on cattle thyroid after the Fukushima Daiichi nuclear power plant accident

**DOI:** 10.1038/s41598-022-25269-0

**Published:** 2022-12-16

**Authors:** Daiki Horikami, Naoya Sayama, Jun Sasaki, Haruka Kusuno, Hiroyuki Matsuzaki, Akane Hayashi, Tatsuro Nakamura, Hiroshi Satoh, Masahiro Natsuhori, Keiji Okada, Nobuhiko Ito, Itaru Sato, Takahisa Murata

**Affiliations:** 1 Department of Animal Radiology, Graduate School of Agriculture and Life Sciences, The University of Tokyo, 1-1-1, Yayoi, Bunkyo-ku, Tokyo, 113-8657 Japan; 2 Department of Veterinary Pathology, Faculty of Agriculture, Iwate University, 3-18-8, Ueda, Morioka, Iwate 020-8550 Japan; 3 MALT (Micro Analysis Laboratory, Tandem Accelerator), The University Museum, The University of Tokyo, 2-11-16, Yayoi, Bunkyo-ku, Tokyo, 113-0032 Japan; 4 Department of Veterinary Pharmacology and Toxicology, Faculty of Agriculture, Iwate University, 3-18-8, Ueda, Morioka, Iwate 020-8550 Japan; 5 Department of Veterinary Radiology, School of Veterinary Medicine, Kitasato University, 35-1, Higashi 23-Bancho, Towada, Aomori 034-0021 Japan; 6 Department of Veterinary Medicine, Faculty of Agriculture, Iwate University, 3-18-8, Ueda, Morioka, Iwate 020-8550 Japan; 7 Food Animal Medicine and Food Safety Research Center, Faculty of Agriculture, Iwate University, 3-18-8, Ueda, Morioka, Iwate 020-8550 Japan

**Keywords:** Zoology, Risk factors, Cancer, Endocrine cancer

## Abstract

Nuclear plant accidents can be a risk for thyroid cancer due to iodine radioisotopes. Near the Fukushima Daiichi nuclear power plant, cattle were exposed to radiation after the accident occurred in May 2011. Here we estimated the total radiation exposure to cattle thyroid and its effects on thyroid function. Until October 2016, the estimated external exposure dose in Farm A was 1416 mGy, while internal exposure dose of ^131^I, ^134^Cs, and ^137^Cs were 85, 8.8, and 9.7 mGy in Farm A and 34, 0.2, and 0.3 mGy in Farm B, respectively. The exposed cattle had thyroid with relatively lower weight and lower level of stable iodine, which did not exhibit any pathological findings. Compared with the control, the plasma level of thyroid-stimulating hormone (TSH) was higher in Farm A cattle born before the accident, while the plasma thyroxine (T4) was higher in Farm A cattle born after the accident, suggesting that exposed cattle showed slight hyperactivation of the thyroid gland. In addition, Farm A cattle have higher level of cortisol, one of the anterior pituitary gland-derived hormones. However, we did not observe a causal relationship between the radiation exposure and cattle thyroid.

## Introduction

Until now, nuclear accident has occurred several times, which causes various types of significant consequences including carcinogenesis (a stochastic effect) including thyroid cancer. For example, the prevalence of all solid nodules, malignant tumors, benign nodules, and cysts in the thyroid was positively correlated with the estimated radiation dose by the atomic bomb in the survivors of Hiroshima and Nagasaki^1,2^. Cardis et al. also performed a case-control study that, in children younger than 15 years of age at the time of the Chernobyl accident, the incidence of thyroid cancer was correlated with the estimated individual doses of radiation exposure^3^. The high risk of thyroid cancer is thought to be caused by the accumulation of radioactive iodine in thyroid follicle. Iodine is necessary for thyroid gland to produce thyroid hormones and usually pooled in the thyroid follicle as thyroglobulin. We humans normally intake and pool commonly present stable iodine (^127^I), while we possibly intake unstable radioactive iodine (^131^I or ^129^I), which is known to be released by nuclear accidents. Compared to ^129^I (a long half-life, 1.57 × 10^7^ years), ^131^I releases rather high nuclear energy with a short half-life (8 days). Cardis et al. showed that the risk of thyroid cancer increased by living in ^127^I-deficient areas and decreased with the administration of potassium iodide, suggested the therapeutic effect of iodine supplementation^3^. Although several studies showed the relationship between radiation exposure and thyroid cancer after the nuclear accidents, a detailed investigation has not been conducted yet.

The accident at the Fukushima Daiichi Nuclear Power Plant (FDNPP) occurred in March 2011. After the accident, people were ordered to evacuate from the ‘deliberate evacuation area’ where was within 20 km from the powerplant and annual cumulative ambient dose rates might exceed over 20 mSv, while many cattle (Japanese Black) in farms were left in the area (Farm A and B; Fig. S1A). These cattle had been internally and/or externally exposed to released radioactive materials, including ^131^I, ^134^Cs, and ^137^Cs for 4–9 years. Since 2013, our group reported the levels of internal and external exposure to radioactive cesium and the results of pathological anatomy of the cattle. Sato et al. detected 3678 ± 618 or 1874 ± 289 Bq/kg of radioactive cesium (total of ^134^Cs and ^137^Cs) in Farm A cattle thyroid (dissected in May or December 2014, respectively)^4^. Sasaki et al. pathologically diagnosed that, among 66 cattle dissected in 2013–2017, there were 3 cattle with goiter and 7 cattle with atrophic thyroid without malignant findings^5,6^. Since there was no correlation between the level of exposure and the pathological changes, we concluded that radiation exposure is not the cause of these diseases. However, we still do not know fully the exposure level of thyroid gland, especially to radioactive iodine, and the effects on thyroid including endocrine function.

In this study, we attempted to estimate the level of internal exposure of the thyroid gland to ^131^I, ^134^Cs, and ^137^Cs and investigate functional changes in the endocrine system, including the thyroid, in cattle living in the ‘deliberate evacuation area’ of the FDNPP accident. In addition, we divided exposed cattle of Farm A and B into two groups; born before the accident (before) or after the accident (after). The exposure situation of them was different because the “before” cattle, but not the “after” cattle, experienced the exposure for first months just after the accident completely. We compared them to the groups of control farms, which we divided by age (younger and older) to try to adjust the age of groups.

## Results

### The ambient dose rate in and around the farm and the external dose of cattle

**Figure 1:**
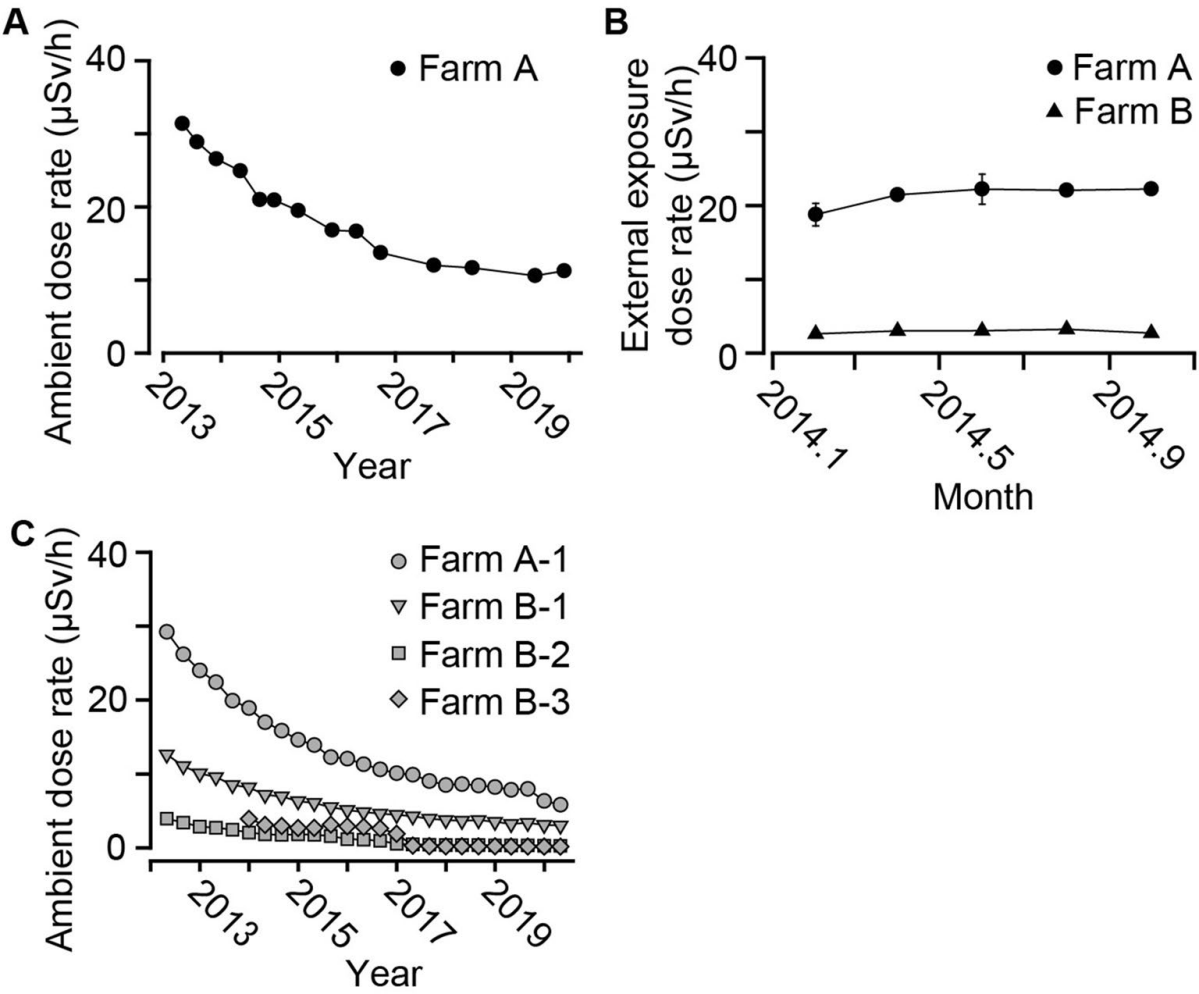
The ambient dose rate in farms and external dose of exposed cattle. The ambient dose rate and external dose of exposed cattle were measured. (**A**) The summary of ambient dose rate at Farm A (black circle, n = 10–73). (**B**) The summary of external dose rate of exposed cattle (Farm A, black circle, n = 17–20, respectively; Farm B, black triangle, n = 11). (**C**) The summary of ambient dose rates around Farm A and B; Farm A-1 (gray circle), B-farm-1 (gray triangle), 2 (gray square), and 3 (gray rhombus). The places of them were shown in Fig. S1B. Data are presented as mean ± SEM.

We measured the ambient dose rate of Farm A (Fig. S1A) within the ‘deliberate evacuation area’ from May 2013 to December 2019, as described previously^5,7^. We used ionization chamber and the dose rate was shown as “Sv”. Before the accident, the ambient dose rate at the ‘deliberate evacuation area’ was 0.040 ± 0.001 µSv/h (Okuma town, Fukushima; measured in 2000–2004)^8^. The ambient dose rate for Farm A was 31.2 ± 0.5 µSv/h in May 2013, and reduced to 11.7 ± 0.1 µSv/h in December 2019 (Fig. 1A). We also measured the external exposure dose rate of the cattle kept within the ‘deliberate evacuation area’ (exposed cattle) by attaching glass badge on the cattle neck^5,7^. The external exposure dose rate of the Farm A cattle (22.2 ± 0.4 μSv/h in August 2014) was similar to the ambient dose rate of Farm A (21.0 ± 0.4 µSv/h in September 2014; Fig. 1B), which was about 7 times higher than that of the Farm B cattle (3.2 ± 0.1 μSv/h in August 2014).

The Japanese government put monitoring posts in Japan, including the ‘deliberate evacuation area,’ from May 2012 (Fig. S1B). The ambient dose rate of the monitoring posts around Farm A were similar to that of Farm A (Farm A-1; 29.2 µSv/h in April 2013, 15.8 µSv/h in August 2014, and 6.3 µSv/h in December 2019) and external exposure of Farm A cattle (Fig. 1C). The ambient dose rate of the monitoring posts around Farm B (Farm B-1, 2, and 3) was also similar (7.0, 1.8, 3.0 μSv/h in August 2014, respectively) to the external exposure of Farm B cattle. These results suggest that the external exposure of Farm A cattle and Farm B cattle were similar to the ambient dose of Farm A and A-1 and Farm B-1, 2, and 3.

As the previous study described^7^, we tried to calculate the cumulative external exposure of Farm A cattle by utilizing the ambient dose of Farm A. Until April 2014 or October 2016 from the accident, the calculated total external exposure in Farm A was 1012 or 1416 mSv, respectively. Considering that the coefficient from Gy to Sv is about 1 in human, the total external exposure of Farm A exposed cattle could be about 1012 or 1416 mGy.

### Physical status of the exposed cattle

**Figure 2:**
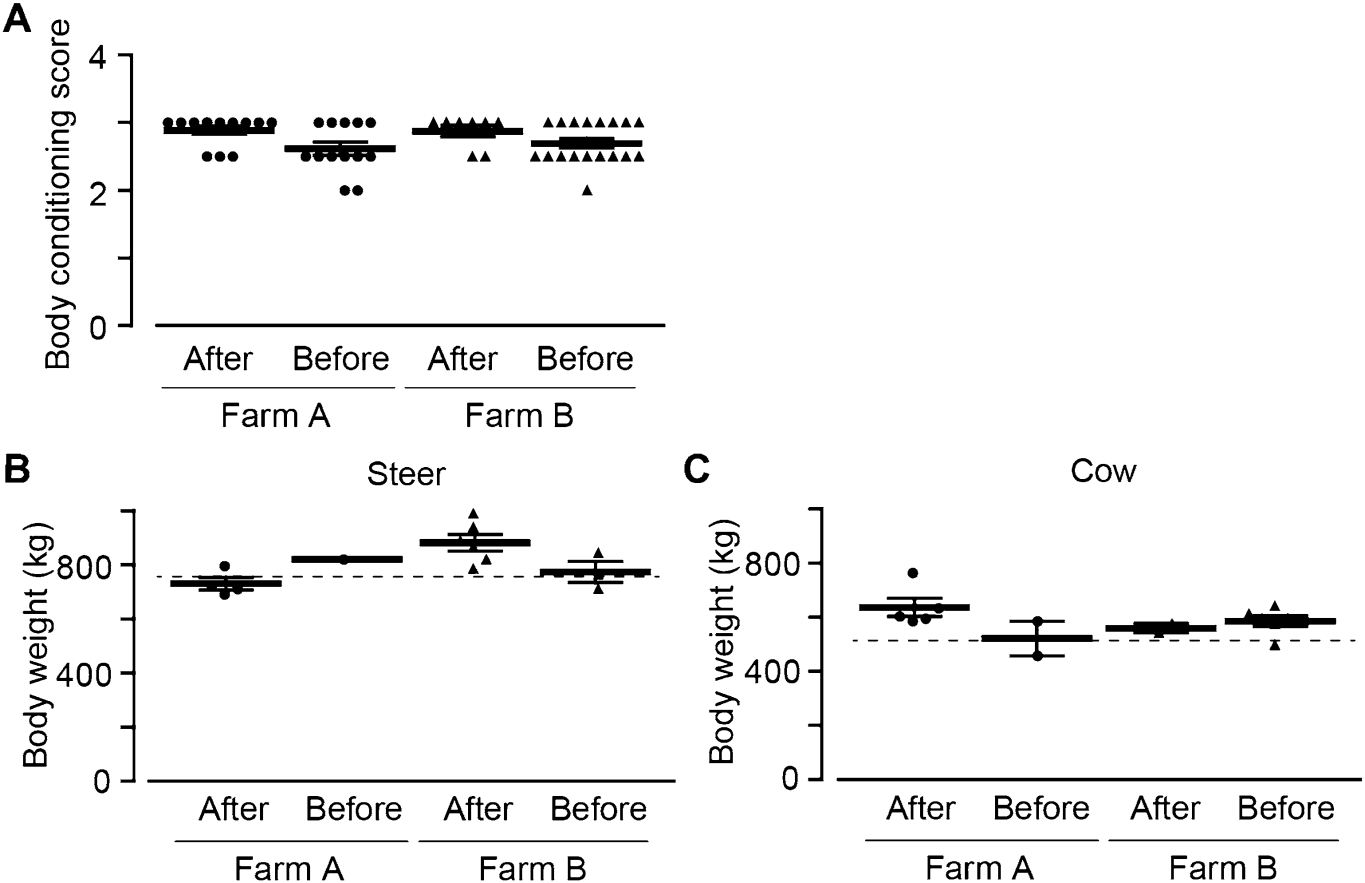
The physique status of exposed cattle. The body conditioning score (BCS) and body weight of exposed cattle were investigated. (**A**) The summary of BCS (Farm A, black circle, n = 14 and 13; Farm B, black triangle, n = 8 and 18). (**B**) The summary of body weight of steer (Farm A, black circle, n = 4 and 1; Farm B, black triangle, n = 6 and 3). (**C**) The summary of body weight of cow (Farm A, black circle, n = 5 and 2; Farm B, black triangle, n = 2 and 6). Broken line, the average weight of Japanese black cattle. Data are presented as mean ± SEM.

We investigated the physical status of the exposed cattle. In December 2018, we took the body conditioning score (BCS) of the exposed cattle in Farm A and B. The standard BCS is between 3 and 3.5. In both farms, there were no cattle with abnormal BCS (over 3.5 or under 1.5) and the BCS of exposed cattle born after the accident was about 2.9 (Fig. 2A; Farm A, 2.89 ± 0.06; Farm B, 2.90 ± 0.10), which was slightly decreased in the cattle born before the accident (Farm A, 2.62 ± 0.10; Farm B, 2.71 ± 0.07). There was no significant difference between Farm A and B. We then investigated the relationship between age and BCS using cattle born before the accident. We could not use cattle born after the accident because we could not identify the detailed birthday after the accident. The age was negatively related to the BCS slightly in Farm A (Fig. S2A) and significantly in Farm B (Fig. S2B).

In addition, we also estimated the body weight of exposed cattle by measuring their body sizes in December 2018. We divided the group by the time of born (before or after) and sex (steer or cow). In Japan, it is known that the average body weight of Japanese Black cattle reaches to 757 kg in steer (5 years old) or 514 kg in cow (2 years old)^9^. Compared to the average weight (broken line), the estimated body weight of steer was similar in Farm A and relatively lower in Farm B (Fig. S2C). The estimated body weight of cow was relatively higher in Farm A and similar in Farm B (Fig. S2D). The aging did not affect the estimated weight of steer or cow (Fig. S2E,F). To confirm the result of body weight, we weighed the body weight of exposed cattle by using a scale in March 2021. The body weight of steer was 729 ± 29 (after) and 820 (before) kg in Farm A and 881 ± 30 (after) and 772 ± 38 (before) in Farm B (Fig. 2B), while that of cow was 635 ± 33 (after) and 521 ± 63 (before) kg in Farm A and 558 ± 18 (after) and 586 ± 20 (before) in Farm B (Fig. 2C). Compared to the average weight (broken line), the weighed body weight of steer and cow was also relatively higher or similar in both farms. There were no cattle with abnormal weight. These results suggest that the exposed cattle did not represent significant physical abnormalities.

### The appearance of the exposed thyroid

**Figure 3:**
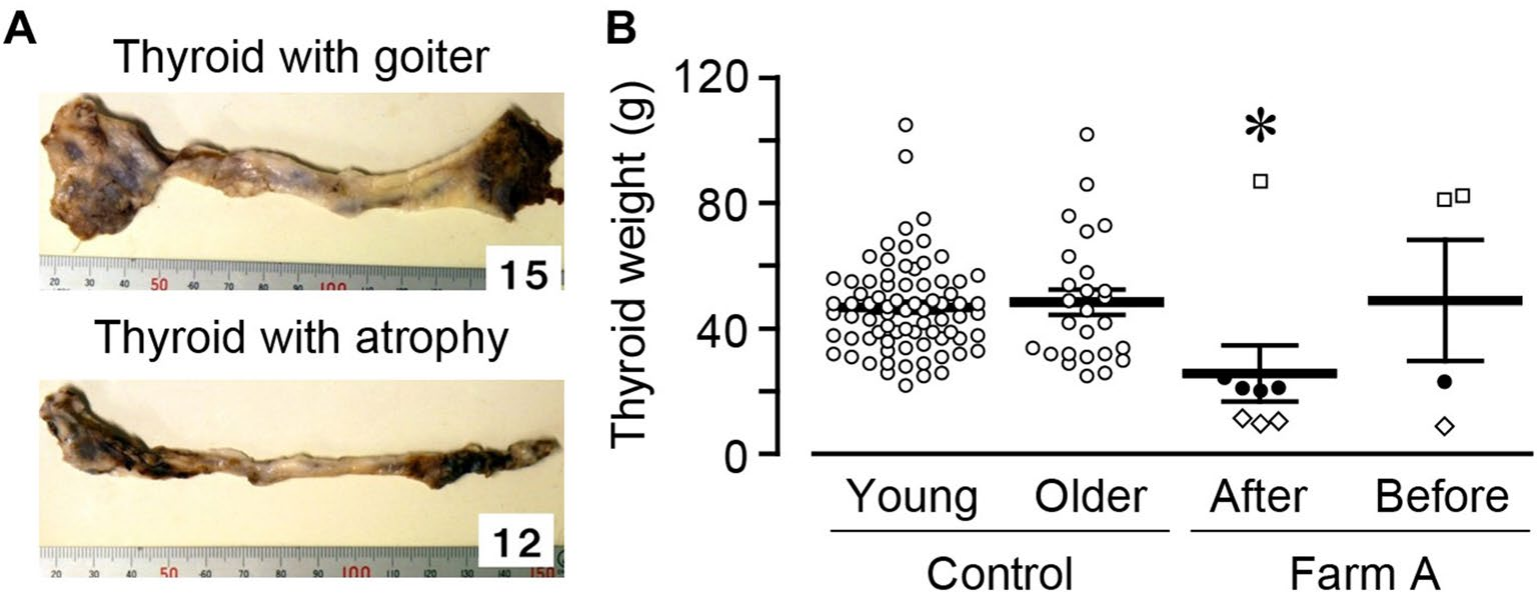
The thyroid weight of exposed cattle. Thyroids were dissected and the weight was measured. (**A**) The representative picture of dissected thyroids with goiter (upper panel) or atrophy (lower panel) in Farm A. Both pictures were taken in October 2013. (**B**) The summary of thyroid weight (Control, open circle, n = 76 and 26; Farm A, black circle (normal), open square (goiter), and open rhombus (atrophic thyroid), n = 8 and 4). Data are presented as mean ± SEM. *P < 0.05 compared with the control group.

As previously described^5,6^, from 2013 to 2017, we excised and pathologically evaluated the thyroid gland of the exposed cattle in Farm A and B. Among 66 necropsy cases, we found 3 cases with goiter (Fig. 3A, upper panel) and 9 cases with atrophic thyroid (lower panel) in Farm A, but no case in Farm B. We here measured the thyroid weight of Farm A cattle by comparing them to control cattle. To adjust to the age of each exposed group (born after or before), we divided control cattle into two groups; younger (young, age less than 3.2 years) and older (age higher than 3.2 years). The thyroid weight in the Farm A cattle born after the accident (dissected in May 2013 and March 2014) were significantly lower than those in the control younger cattle (in May 2017, Fig. 3B). The thyroid weight of Farm A before cattle (in March 2014, April 2014, and September 2014) was not significantly changed compared with that in control older cattle and Farm A after cattle. The weight of thyroid with goiter was 83.6 ± 1.7 g (open circle; in March 2013, April 2014, and September 2014), while that of atrophic thyroid was 10.2 ± 0.5 g (open rhombus; in March 2014). We did not find the relationship between the age and the thyroid weight (Fig. S3A,B).

We previously diagnosed these cases of goiter and atrophic thyroid^6^. Pathological study showed that, in thyroid with goiter, there was no mass or nodule lesion. These thyroids with goiter showed diffuse proliferation of follicular epithelium; many follicles showed normal appearance, while some follicles had mild papillary projections formed by hyperplastic columnar epithelial cells into the lumen. There was no malignant finding including cellular atypia, mitotic figures, or capsular invasion. In addition, all atrophic thyroids did not show any findings of inflammation such as cell infiltration and stromal fibrosis. In addition, we did not find any positive signaling for Ki-67 (cell proliferation), 8-nitroguanosine (oxidative stress), or TUNEL (cell apoptosis) in both cases of goiter and atrophic thyroid.

### The estimated internal dose of exposed thyroid to ^131^I

**Figure 4:**
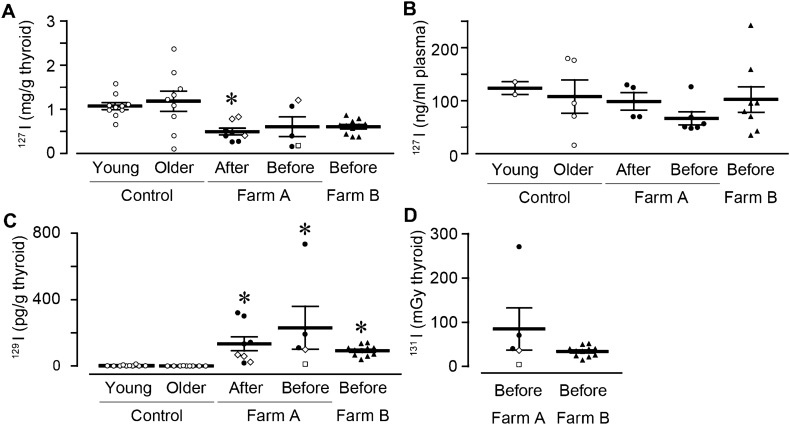
Internal exposure dose by ^131^I in cattle thyroid. Thyroids were dissected and the concentrations of ^127^I and ^129^I were measured. (**A**) The summary of thyroid ^127^I (Control, open circle, n = 10 and 9; Farm A, black circle (normal), open square (goiter), and open rhombus (atrophic thyroid), n = 8 and 5; Farm B, black triangle (normal), n = 11). (**B**) The summary of plasma ^127^I (Control, open circle, n = 2 and 5; Farm A, black circle (normal), open square (goiter), and open rhombus (atrophic thyroid), n = 4 and 6; Farm B, black triangle (normal), n = 8). (**C**) The summary of thyroid ^129^I (Control, open circle, n = 10 and 9; Farm A, black circle (normal), open square (goiter), and open rhombus (atrophic thyroid), n = 8 and 5; Farm B, black triangle (normal), n = 11). (**D**) The summary of estimated thyroid ^131^I from the time of the FNPP accident to the euthanasia (Farm A, black circle (normal), open square (goiter), and open rhombus (atrophic thyroid), n = 5; Farm B, black triangle (normal), n = 11). Data are presented as mean ± SEM. *P < 0.05 compared with the control group.

Using the dissected thyroids, we investigated the internal exposure of the thyroid to radioactive iodine (^131^I). We initially measured the concentration of ^127^I, a stable isotope of iodine by ICP-MS system. The concentration of thyroid ^127^I were about 2 times lower significantly in Farm A cattle born after the accident (0.50 ± 0.08 mg/g, dissected in March 2014 and May 2014) compared to that in control younger cattle (1.08 ± 0.08 mg/g in May 2017; Fig. 4A). The thyroid ^127^I in Farm B before (0.61 ± 0.11 mg/g in June 2016, November 2016, and April 2017) and Farm A before (0.60 ± 0.22 mg/g; in March 2014, September 2014, and October 2016) similarly showed relatively lower thyroid ^127^I concentration compared with that in control older cattle (1.19 ± 0.23 mg/g). Since thyroid gland is known to keep iodine, this result suggested that exposed cattle in Farm A and B lack iodine, there was no significance in plasma level of ^127^I between the control farms and Farm A or B (Fig. 4B). There was no relationship between the age and the level of ^127^I in thyroid (Fig. S4A–C) or plasma (Fig. S4D–F).

We next measured the concentration of ^129^I, a long-lived iodine radioisotope instead of ^131^I (short-lived iodine radioisotope) using the AMS system. The concentration of thyroid ^129^I was about 100 times higher in the exposed thyroid (Fig. 4C; Farm A after, 134 ± 42 pg/g; Farm A before, 230 ± 129 pg/g; Farm B before, 92 ± 9 pg/g) than in the control thyroid (2.7 ± 1.2 and 0.7 ± 0.3 pg/g). The concentration of ^129^I in the thyroid with goiter was relatively low (open square, 12 pg/g). We did not find any effect of aging on thyroid ^129^I level (Fig. S4G–I). Using this data, since almost exposure to ^131^I occurred several months after the FDNPP accident, we estimated the total internal exposure of the thyroid gland to ^131^I in exposed cattle born before the accident. The calculated level of internal exposure of the thyroid gland to ^131^I was 85 ± 48 mGy for Farm A and 34 ± 4 mGy for Farm B (Fig. 4D).

We investigated the effect of sampling time on the concentration of thyroid or plasma ^129^I. The cattle in Farm A ate local grass and straw during summer (May to October), and concentrated feed in winter (November to April). The cattle in Farm B ate concentrated feed all year round. In Farm A, the concentration of ^127^I in the local grass, the straw, the concentrated feed, and the mineral salt were 42.8, 56.6, 8.14, and 344 ng/g, respectively (Fig. S5A). The concentration of ^129^I were much higher in local grass (2.41 × 10^−3^ pg/g), straw (0.96 × 10^−3^ pg/g) than in mineral salt (0.01 × 10^−3^ pg/g) and concentrated feed (not detected; Fig. S5B), suggesting that the exposed cattle in Farm A mainly ingested ^129^I during summer. Accordingly, as shown in Fig. S5C, the concentration of ^129^I was higher during summer (in May 2014, September 2014, and October 2016) than during winter (in March 2014) in Farm A cattle. In Farm B, the concentrations of ^129^I during summer (in June 2016) and winter (in November 2016 and April 2017) were similar. Since it is known that radioactive substances are gradually diluted by windows and rain in natural environments; called weathering effects, we measured the concentration of plasma ^129^I during the summer. In both Farm A and B, the concentration of plasma ^129^I declined by year (Fig. S5D).

### The estimated internal dose of exposed thyroid to ^134^Cs and ^137^Cs

As previously measured^10^, we investigated the concentrations of the cesium radioisotopes (^134^Cs and ^137^Cs) in cattle thyroid and plasma to estimate the extent of internal exposure to ^134^Cs and ^137^Cs.

**Figure 5:**
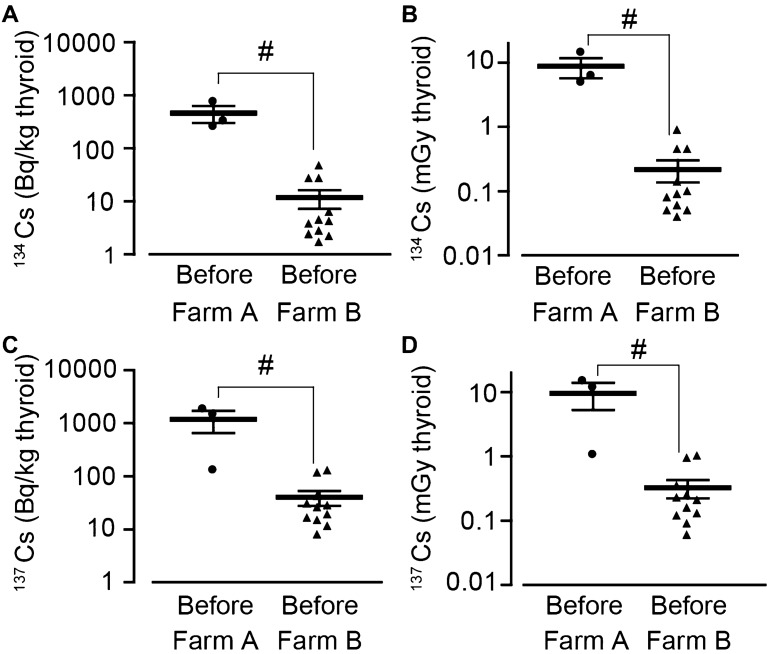
Internal exposure dose by ^134^Cs and ^137^Cs in cattle thyroid. Thyroids were dissected and the levels of ^134^Cs and ^137^Cs were measured. (**A**,**B**) The summary of thyroid ^134^Cs (Farm A, black circle, n = 3; Farm B, black triangle, n = 11), shown as (**A**) the becquerel and (**B**) the calculated internal exposure dose until October 2016. (**C**,**D**) The summary of thyroid ^137^Cs (Farm A, black circle, n = 3; Farm B, black triangle, n = 11), shown as (**C**) the becquerel and (**D**) the calculated internal exposure dose until October 2016. Data are presented as mean ± SEM. ^#^P < 0.05.

The concentration of thyroid ^134^Cs was higher Farm A cattle born before the accident (466 ± 163 Bq/kg, dissected in October 2016) than Farm B before cattle (11.8 ± 4.6 Bq/kg in June 2016, November 2016, and April 2017; Fig. 5A). Similarly, the concentration of the plasma ^134^Cs for Farm A was higher than that for Farm B (Fig. S6A). The aging did not significantly affect the level of thyroid ^134^Cs (Fig. S6B,C). The calculated internal exposure to ^134^Cs until October 2016 was 8.8 ± 3.0 mGy (Farm A before) and 0.2 ± 0.1 mGy (Farm B before; Fig. 5B), while that until April 2014 was 6.7 ± 2.3 and 0.2 ± 0.1 mGy, respectively (data not shown).

In addition, the concentrations of thyroid ^137^Cs were also higher in Farm A before (1190 ± 539 Bq/kg) than in Farm B before (40.4 ± 12.8 Bq/kg; Fig. 5C). The concentration of plasma ^137^Cs for Farm A was also higher than that for Farm B (Fig. S6D). There was no relationship between the age and the level of thyroid ^137^Cs (Fig. S6E,F). The calculated internal exposure to ^137^Cs until October 2016 was 9.6 ± 4.4 mGy (Farm A before) and 0.3 ± 0.1 mGy (Farm B before; Fig. 5D), while that until April 2014 was 5.5 ± 2.5 and 0.2 ± 0.1 mGy, respectively (data not shown).

From these results, the total internal exposure of the thyroid gland to ^134^Cs and ^137^Cs until April 2014 or October 2016 was 13.2 or 18.5 mGy in Farm A and 0.4 or 0.5 mGy in Farm B. From the result that estimated the level of the internal/external exposure of cattle, we can estimate that total exposure of Farm A cattle which born before the accident was 1110 mGy until April 2014 and 1520 mGy until October 2016 (Table 1).

**Table 1:** The total exposure of cattle thyroid in Farm A and B.

Dissected time		Farm A		Farm B	
		Apr. 2014	Oct. 2016	Apr. 2014	Oct. 2016
External exposure (mGy)		1012	1416	–	–
Intenal exposure (mGy)	^131^I	85 ± 48	85 ± 48	34 ± 4	34 ± 4
	^134^Cs	6.7 ± 2.3	8.8 ± 3.0	0.2 ± 0.1	0.2 ± 0.1
	^137^Cs	5.5 ± 2.5	9.6 ± 4.4	0.2 ± 0.1	0.3 ± 0.1
Total exposure (mGy)		1110	1520	–	–

### The level of plasma thyroid hormones in exposed cattle

Thyroid is known to keep homeostasis by producing triiodothyronine (T3) and thyroxine (T4), which production is promoted by thyroid-stimulating hormone (TSH). These thyroid hormones are known to represent the status of thyroid function. Here, we investigated the effect of the exposure on thyroid function by measuring the concentrations of these thyroid hormones in Farm A cattle plasma in 2014-2016. For the investigation of plasma hormones, we divided the control cattle again into two groups; younger (young, age less than 5.2 years) and older (age higher than 5.2 years).

**Figure 6:**
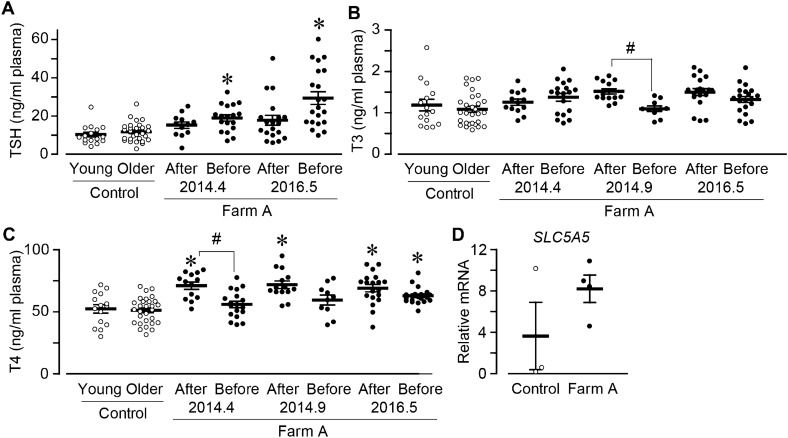
The level of thyroid hormones in plasma. (**A**–**C**) Plasma was collected in 2014–2016 and the levels of thyroid hormones were measured. The summary of (**A**) TSH (Control, open circle, n = 15 and 29; Farm A, black circle, n = 13, 18, 19, and 21), (**B**) T3 (Control, open circle, n = 15 and 29; Farm A, black circle, n = 14, 18, 14, 10, 19, and 21), and (**C**) T4 (Control, open circle, n = 15 and 29; Farm A, black circle, n = 14, 18, 14, 10, 19, and 21). (**D**) The summary of thyroid mRNA level of NIS (*SLC5A5*; control, open circle, n = 3; Farm A, black circle, n = 4). Data are presented as mean ± SEM. *P < 0.05 compared with the control group. ^#^P < 0.05.

The concentration of plasma TSH was slightly, but not significantly higher in cattle born after the accident (15.0 ± 1.6 ng/ml, taken in April 2014; 17.8 ± 2.6 ng/ml in May 2016) than those in the control younger cattle (10.3 ± 1.2 ng/ml in April-August 2017; Fig. 6A). On the other hand, the plasma TSH in cattle before was significantly higher (19.0 ± 1.6 ng/ml and 29.4 ± 3.3 ng/ml) compared with that in the control older cattle (11.7 ± 1.0 ng/ml). The concentration of TSH was slightly higher in cattle before than that after. Although TSH concentration is known to be increased by aging, the plasma TSH in control and Farm A cattle was increased slightly, but not significantly by the aging (Fig. S7A–C). The concentrations of plasma T3 were tended to be higher in the Farm A cattle born after and before the accident (1.5 ± 0.1 and 1.3 ± 0.1 ng/ml in May 2016; Fig. 6B) than that of the control younger and older cattle (1.2 ± 0.1 and 1.1 ± 0.1 ng/ml). In only September 2014, there was a difference between the cattle after and before. The plasma T3 is known to be decreased by age as opposed to TSH. The investigation of the effect of age showed that the plasma T3 level had a significant negative correlation to the age in Farm A cattle (Fig. S7D–G). The plasma T4 concentrations for the cattle born after at all time point were significantly higher than that in the control younger cattle (71.0 ± 2.9 ng/ml in April 2014; 71.8 ± 3.0 ng/ml in September 2014; 69.0 ± 3.0 ng/ml in May 2016; control younger, 52.3 ± 3.4 ng/ml, Fig. 6C). The plasma T4 was also higher in the cattle before than control older in May 2015 (62.9 ± 1.4 ng/ml in May 2016; control older, 51.2 ± 2.0 ng/ml). In only April 2014, the plasma T4 was significantly lower in the cattle before (56.0 ± 2.6 ng/ml) than that after in April 2014. There was no relationship between the age and plasma T4 (Fig. S7H–K). We then measured the thyroid expression level of the sodium/iodide cotransporter (NIS), which transports iodine from blood flow into cytosol and is needed for thyroid hormone production. The exposed thyroid tissue had relatively, but not significantly higher mRNA levels of NIS (*SLC5A5*, dissected in May 2014; Fig. 6D).

These results indicate that exposed cattle had the higher concentrations of thyroid hormones, especially T4, and cattle born before the accident had higher level of TSH and lower level of T3 and T4, suggesting slight hyperactivation in thyroid gland.

### The level of plasma anterior pituitary-derived hormones in exposed cattle

Although T3 and T4 are known to have negative feedback action on TSH levels, plasma TSH was higher in exposed cattle as well as plasma T3 and T4. Since anterior pituitary produces TSH, we then investigated the effect of ionizing radiation on the anterior pituitary by measuring anterior pituitary-derived hormones; cortisol, growth hormone (GH), and insulin-like growth factor-1 (IGF-1).

**Figure 7:**
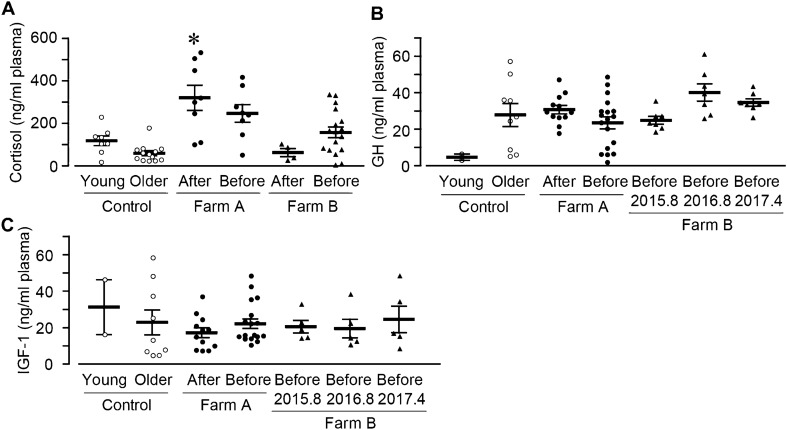
The concentration of anterior pituitary-derived hormones in plasma. Plasma was collected and the levels of anterior pituitary-derived hormones were measured. The summary of (**A**) cortisol (Control, open circle, n = 8 and 13; Farm A, black circle, n = 8 and 9; Farm B, black triangle, n = 4 and 17), (**B**) GH (Control, open circle, n = 2 and 9; Farm A, black circle, n = 14 and 18; Farm B, black triangle, n = 7 each), and (**C**) IGF-1 (Control, open circle, n = 2 and 9; Farm A, black circle, n = 14 and 18; Farm B, black triangle, n = 5 each). Data are presented as mean ± SEM. *P < 0.05 compared with the control group.

The plasma cortisol concentrations were significantly higher in the Farm A cattle born after, but not before the accident (after, taken in September 2014 and October 2016; before, in May 2013, May 2014, September 2014, and March 2017). There was no significant difference in Farm B (after, in May 2016; before, in May 2016 and April 2017) compared with that in control (younger and older, in April-August 2017; Fig. 7A). The relationship analysis showed that plasma cortisol was decreased by age in control farms, while increased in Farm B, but not in Farm A (Fig. S8A–C). On the other hand, the concentration of plasma GH and IGF-1 were not changed among control cattle (in August 2017, April 2017, May 2017, and June 2017), Farm A cattle (in September 2014), and Farm B cattle (in August 2015, August 2016, and April 2017; Fig. 7B,C), which was not affected by the age (Fig. S8D–M). Furthermore, all plasma anterior pituitary-derived hormones were not changed between cattle born before and after the accident.

These results suggest that ionizing radiation increases the concentration of corticoid in Farm A.

## Discussion

Although it is known that nuclear accidents represent a significant concern globally, we still need to carefully assess the effect of ionizing radiation on the body including thyroid gland. In the present study, we estimated the levels of internal and external radiation exposure and investigated their effects on the thyroid gland including endocrine function in exposed cattle, which had been kept in two farms with different radiation doses within the ‘deliberate evacuation area’ for several years after the FDNPP accident (2011–2017). The exposed cattle (total exposure was 1110–1520 in Farm A) had lower weight of thyroid and lower ^127^I. Plasma analysis showed that the plasma concentrations of both TSH and T4 in the exposed cattle. We also found higher plasma concentrations of cortisol, one of anterior pituitary-derived hormones. However, considering the internal and external radiation concentrations and periods, we did not observe an obvious relationship between radiation exposure and pathological/functional changes in the cattle thyroid gland.

Several studies investigated the thyroid dose of evacuees from the nuclear power plant accident. In the residents who were evacuated after the FDNPP accident on 11th March 2011, the median thyroid dose from 12th to 16th April 2011 to 15th May 2011 was 3.5–4.2 mSv^11^. In the Chernobyl accident in 26th April 1986, the average thyroid dose of the residents (Pripyat, Ukraine) evacuated within 40 h was 370 mSv, which was estimated using NaI (Tl) scintillation spectrometers^12^. The United Nations Scientific Committee on the Effects of Atomic Radiation (UNSCEAR) also reported that, in Chernobyl accident, the average thyroid dose of evacuee (during April-September in 1986) from Belarus, Russia, and Ukraine are 1077, 440, and 333 mSv, respectively^13^. Although we need to note the contradiction between humans and cattle, presumably the cattle thyroid in this study exposed to higher dose (1110–1520 mGy) compared with these evacuees.

This estimated total exposure dose may be an underestimate of the actual exposure dose. Since the physical half-life of ^129^I is 5.73 × 10^9^ days, we hypothesized that the dose of ^129^I did not change after the accident. However, radioactive substances are gradually diluted by windows and rain in natural environments; called weathering effects. Indeed, the ambient dose reflecting environmental radioactive substances in the farms decreased yearly. The plasma I^129^ concentration, which was presumably equivalent to environmental ^129^I in cattle, was also decreased yearly. Thus, the actual exposure dose of cattle can be higher than our current estimation. In addition, while we here focused on the effects of ^131^I, ^134^Cs, and ^137^Cs, we did not include other types of radioactive iodine (^132^I) and radioactive tellurium (^129m^Te, ^129^Te, and ^132^Te) in the estimation of internal exposure. The actual exposure dose can be higher than our estimation.

Although A farm is farther to FNPP than B farm, the level of external exposure was 10 times higher in A farm than that in B farm. This difference probably came from the wind direction. Several days after the FDNPP accident, radioactive materials moved northwest on the wind and then accumulated on the soil by rain. Indeed, previous report showed that air dose was high in the northwest of the nuclear powerplant^14^.

Since radioactive ^131^I decays rapidly (physical half-life: 8.02 days), researchers often estimate the level of internal exposure to ^131^I based on an index that is easy to obtain. Kim et al. estimated the internal exposure level to ^131^I of people after an FDNPP accident using the committed effective dose of ^137^Cs^15^. They utilized the emission ratio of ^131^I/^137^Cs and estimated that the internal exposure level to ^131^I was approximately 34 mGy. We estimated the internal exposure level of the cattle thyroid gland to ^131^I (Farm A; 85 ± 48 mGy, Farm B; 34 ± 4 mGy) using the emission ratio of ^131^I/^129^I (0.033)^16^. Considering the different living environments of humans and cattle, these doses can be reasonable.

We showed that the average thyroid weight of exposed cattle was lower than that of control cattle. This result is consistent with previous reports in human that ionizing radiation induces thyroid atrophy, but not only thyroid cancer or nodule formation. Ostroumova et al. reported that 2 Gy radiation exposure reduced the thyroid gland in Belarusian children after the Chernobyl accident^17^. Shir et al. also showed a clinical study that radiation therapy reduces the thyroid glands^18^. However, there are still some concerns as to whether these changes (including goiter or atrophy) are caused by radiation in this study. As previously reported, that all cases with goiter and atrophy exhibit no nodular regions, cystic follicles, malignant findings, or inflammatory findings^6^. Cells with DNA damage or apoptosis was not observed either. Furthermore, we here showed that, although the data size is small (n=1 each), thyroid with goiter or atrophy had relatively lower level of ^129^I compared with other thyroids in Farm A. Further investigations are required to reveal the relationship between thyroid with goiter or atrophy and ionizing radiation.

The concentration of stable iodine ^127^I in the thyroid gland was lower in cattle in both farms A and B than in the control farms, suggesting iodine deficiency in exposed cattle. It is known that iodine deficiency is a common cause of goiter. However, Japanese soil surrounded by the sea contains sufficient iodine and the cattle we used in the present study could take enough iodine from mineral salts and water. Indeed, the concentrations of plasma iodine in cattle from farms A and B and the control farms were comparable. There is a possibility that environmental changes somehow influence iodine storage of the thyroid gland. We also cannot explain the discrepancy that some cattle have goiter even though they retained less iodine in their thyroid.

Here, the concentrations of plasma TSH and T4 were significantly higher in the exposed cattle than in the control cattle. The plasma T3 in the exposed cattle was also slightly higher. Alawneh et al. showed a similar result that the medical exposure of radiology technologists increases the concentrations of plasma TSH, T3, and T4^19^. To clarify whether ionizing radiation upregulates T3 and T4 production by activating anterior pituitary and increasing TSH production, we measured plasma anterior pituitary-derived hormones; corticoid, GH, and IGF-1. The concentration of cortisol was higher in the exposed cattle, while GH or IGF-1 was not higher. Considering that the living environments and the nutritional status of cattle differ greatly between exposed cattle (grazed in the farm) and control cattle (bred under human management), the stress associated with the lack of care and the condition of the food affects, but not radiation-induced anterior pituitary, increased the level of cortisol in exposed cattle. The mechanism by which ionizing radiation increases these hormones is still unknown.

It is known that age affects the prevalence of radiation-induced thyroid cancer. After the Chernobyl disaster, a significant increase in the prevalence of thyroid cancer was reported among children and adolescents exposed to radioactive iodine released at the time of the accident in Belarus, Russia, and Ukraine^13^. Imaizumi et al. also showed that the radiation dose-response was higher in atomic bomb survivors who were exposed when they were young (mean median thyroid radiation doses of all survivors, 449 mSv)^2^. However, the other effects of age on the exposed thyroid is still unclear. Here we showed that the case of goiter and atrophy were found in cattle both born after and before. In addition, in Farm A cattle born before the accident, the age of cattle was not significantly related to the concentration of thyroid ^127^I, ^129^I, ^134^Cs, and ^137^Cs and plasma Cortisol, GH, and IGF-1. On the other hand, in some of time points, the plasma concentration of T3 and T4 in cattle before the accident were lower compared to that in cattle after. The cattle born before the accident (9.2 ± 0.7 years) were older than those born after the accident (3.5 ± 0.1 months). It is known that aging increases the concentration of TSH and decreases those of T3 and T4^20^. Consistently, the plasma T3 of Farm A cattle was negatively correlated to the age. The differences of thyroid hormones between the time of born were caused presumably by age rather than the effect of radiation exposure.

We here tried to investigate the effect of chronic radiation exposure on the thyroid gland in cattle with focusing on thyroid function. It was meaningful as it has shown the level of internal exposure to ^131^I in thyroid tissue in association with the whole-body status of the exposed cattle. In addition, cattle with relatively long-life span were useful animals for this study. We would like to continue the investigations in the hope that similar accidents will never happen again.

## Methods

### Samples

All animal experiments and methods were approved by the Institutional Animal Care and Use Committee of the University of Tokyo and the animal experiment committee of Iwate University (Permit No. P-180427004). This study was reported in accordance with ARRIVE guidelines.

We used Japanese Black cattle kept in Farm A (Namie town, Fukushima, closed circle) and B (Okuma town, Fukushima, closed triangle; Fig. S1A). We divided the exposed cattle into two groups; cattle born after the accident and before. The cattle born before the accident experienced the first months after the accident, while cattle after did not. Although we could not identify the certain birthday of cattle born after the accident, we did not use calves which age was under 1 year old. As control, we used Japanese Black cattle without calves kept in Japanese farms; Hokkaido, Aomori, Miyagi, Iwate, Tottori, and Kumamoto (National Agriculture and Food Research Organization). We also divided the control cattle into two groups; younger (young) cattle and older cattle. To adjust the age of control groups to exposed groups, we defined the cattle under 3.2 years old as “younger” for thyroid experiments, while the cattle under 5.2 years old as “younger” for plasma experiments. The list of thyroid and plasma samples were shown in Tables S1 and S2.

### Measurement of ambient dose rate (external exposure)

The ambient dose rate in Farm A was measured by putting ionization chamber (ICS-323C, Hitachi ALOKA, Tokyo, Japan) 1 m above the ground at the multiple points of the farm from May 2013 to September 2017. The data of dose rate in Farm A was published previously in our study^7^.

The ambient dose rate around Farm A and B was measured with a monitoring post (Fig. S1B, Nuclear Regulation Authority, http://radioactivity.nsr.go.jp/map/ja/) from April 2012 to April 2020.

### Measurement of external exposure rate

The glass badge (Chiyoda Technol Corporation, Tokyo, Japan) was attached to the neck of cattle for 1-4 months and the absorbed dose of the attached badge was measured by manufacture manner. The external exposure level was calculated by dividing the absorbed dose by the time of attachment.

### Measurement of body conditioning score (BCS)

We obtained the BCS of cattle by assessing the covering fat around the tailhead area as described previously^21,22^; 1.5, the line between anus and vulva tilt to 45 degrees; 2, the vertical depressions on both sides of anus or the prominent tailhead; 2.5, the horizontal depressions on both sides of the anus; 3, the smooth tailhead; 3.5, the flat tailhead; 4, the rounded tailhead.

### Estimation of body weight

We measured chest girth (CG), body length (BL), and hip height (HH) of cattle. The body weight of cattle were estimated by using the formula which was established as described previously^23^.

$${\text{Body weight }}\left( {{\text{kg}}} \right) \, = { 3}.{7 } \times {\text{ CC }} + { 2}.{4 } \times {\text{ BL }} + { 2}.{3 } \times {\text{ HH }} - { 862}.{2}$$

### Dissection of thyroid gland

In May 2014-June 2017, the cattle in Farm A and B (Table S1) were anesthetized with xylazine (0.2 mg/kg, intramuscularly) and pentobarbital (20 mg/kg, intravenously) and then euthanized with suxamethonium (2 mg/kg, intravenously) or barbiturate/saturated KCl solution, which followed the American Veterinary Medical Association euthanasia guidelines and were performed with the owners’ consent. After the euthanasia, the autopsies were performed by the veterinarian, following the Institutional Animal Care and Use Committee, and approved by the President of Iwate University (permit number: A201438). After the autopsies, the thyroid was isolated, and the wet weight of the thyroid was measured. The thyroids in the control farms (Table S1) were obtained from a slaughterhouse in Iwate Prefecture in May 2017. The data of thyroid weight in Farm A and control farms was published previously in our study^6^.

### Collect blood sample

Blood of the cattle in Farm A, Farm B, and control farms was collected from April 2014 to June 2017 (Table S2). Whole blood samples were used for radioactive cesium measurement. To obtain a plasma sample, collected blood was incubated in BD VACUTAINER Heparin Tubes and then centrifugated (3000 rpm, 4 °C, 15 min). The supernatant was collected as a plasma sample and used for the measurement of the iodine and hormones.

### Measurement of the level of ^127^I and ^129^I

We measured the level of ^127^I and ^129^I as described previously^24^. The aliquot freeze-dried sample was combusted and then trapped in an alkaline solution containing 2% tetramethylammonium hydroxide and 0.005% Na_2_SO_3_.

Accelerator mass spectrometry (AMS) analysis was carried out at Micro Analysis Laboratory, Tandem Accelerator, the University of Tokyo (MALT). For only AMS analysis, trapped iodine was purified as AgI. ^129^I/^127^I atomic ratio was normalized by Z94-0596 from Prime Laboratory, Purdue University (^129^I/^127^I atomic ratio = 6.540 × 10^−11^^25^).

Quantitative analysis of stable iodine (^127^I) was carried out with ICP-MS (Agilent 7500a, Agilent technology, CA, USA). ^133^Cs solution was used as a standard solution. ^127^I results were normalized by results of ^133^Cs internal standard, and ^127^I concentration was calculated with calibration curves.

### The estimation of internal exposure by ^131^I

As shown in the graph (Fig. S9), ingested ^131^I was accumulated and then discharged by the physical half-life (8.02 days), while ingested ^129^I was accumulated and then reached equilibrium concentration by its long half-life (5.73 × 10^9^ days). Assuming the molecular emission ratio of ^129^I and ^131^I (^129^I:^131^I = 30:1)^16,26^ and the biological half-life of iodine in cattle (100 days), we initially calculated the daily uptake of thyroid ^131^I immediately after the accident (D_0(131)_) by this formula shown in below:

$$\begin{gathered} {\text{C}}_{{{\text{sam}}({129})}} = {\text{ D}}_{{{\text{sam}}({129})}} /{\text{ Kel}}_{{({129})}} \times \, \left( {{1 }{-}{\text{ e}}^{{{-}{\text{kel}}\left( {{129}} \right){\text{t}}}} } \right) \hfill \\ {\text{C}}_{{{\text{sam}}({129})}} = {\text{ D}}_{{0({129})}} /{\text{ Kel}}_{{({129})}} \left( {{\text{D}}_{{{\text{sam}}({129})}} = {\text{ D}}_{{0({129})}} ,{\text{ t }} > > {\text{ Te}}_{{({129})}} } \right) \hfill \\ {\text{C}}_{{{\text{sam}}({129})}} = \, \left( {{\text{D}}_{{0({131})}} \times { 3}0} \right) \, /{\text{ Kel}}_{{({129})}} \left( {{\text{D}}_{{0({131})}} = {\text{ D}}_{{0({129})}} /{ 3}0} \right) \hfill \\ {\text{D}}_{{0({131})}} = {\text{ C}}_{{{\text{sam}}({129})}} \times {\text{ Kel}}_{{({129})}} /{ 3}0 \hfill \\ {\text{t}}:{\text{ from the accident to the euthanasia }}\left( {{\text{day}}} \right) \hfill \\ {\text{C}}:{\text{ concentration of thyroid}}^{{\text{129 or 131}}} {\text{I }}\left( {{\text{ng}}/{\text{kg}}} \right) \hfill \\ {\text{C}}_{{{\text{sam}}({129})}} :{\text{ concentration of}}^{{{129}}} {\text{I at the sampling time }}\left( {{\text{pg}}/{\text{g }} = {\text{ ng}}/{\text{kg}}} \right) \hfill \\ {\text{D}}:{\text{ daily uptake of thyroid}}^{{\text{129 or 131}}} {\text{I }}\left( {{\text{ng}}/{\text{kg}}/{\text{day}}} \right) \hfill \\ {\text{D}}_{0} :{\text{ daily uptake of thyroid}}^{{\text{129 or 131}}} {\text{I immediately after the accident }}\left( {{\text{ng}}/{\text{kg}}/{\text{day}}} \right) \hfill \\ {\text{Kel}}:{\text{effective decay constant of}}^{{\text{129 or 131}}} {\text{I}} = \, 0.00{\text{693 or }}0.0{934 }\left( {/{\text{day}}} \right) \hfill \\ {\text{Te}}:{\text{ effective half}} - {\text{life of thyroid}}^{{\text{129 or 131}}} {\text{I }} = { 1}00{\text{ or 7}}.{42 }\left( {{\text{day}}} \right) \hfill \\ \end{gathered}$$

Then, we calculated the accumulated internal dose of ^131^I (U_accum_) from the time of accident to October 2016 by this formula shown in below:

$$\begin{gathered} {\text{C}}_{{{\text{sam}}({131})}} = {\text{ D}}_{{0({131})}} / \, \left( {\lambda \, {-}{\text{ Kel}}_{{({131})}} } \right) \, \times \, \left( {{\text{e}}^{{{-}{\text{Kel}}\left( {{131}} \right){\text{t}}}} {-}{\text{ e}}^{{{-}\lambda {\text{t}}}} } \right) \hfill \\ {\text{U}}_{{({131})}} \left( {{\text{nGy}}/{\text{day}}} \right) \, = {\text{ C}}_{{({131})}} \times {\text{ SA }} \times {\text{ DCF}} \hfill \\ {\text{U}}_{{{\text{accum}}({131})}} \left( {{\text{mGy}}} \right) \, = \, \smallint {\text{ U}}_{{({131})}} {\text{dt}} \hfill \\ \lambda_{{({131})}} :{\text{ disintegration constant of}}^{{{131}}} {\text{I }} = \, 0.0{864 }\left( {/{\text{day}}} \right) \hfill \\ {\text{Tb}}:{\text{ biological half}} - {\text{life of iodine in thyroid }} = { 1}00 \, \left( {{\text{day}}} \right) \hfill \\ {\text{A}}_{{({131})}} :{\text{ radioactivity of thyroid}}^{{{131}}} {\text{I }}\left( {{\text{Bq}}/{\text{kg}}} \right) \hfill \\ {\text{SA}}_{{({131})}} :{\text{ specific activity of}}^{{{131}}} {\text{I }} = { 4}.{6 }\left( {{\text{MBq}}/{\text{ng}}} \right) \hfill \\ {\text{DCF}}_{{({131})}} :{\text{ dose conversion factor of}}^{{{131}}} {\text{I }} = { 2}.{8 }\left( {{\text{nGy}}/{\text{day}}/\left( {{\text{Bq}}/{\text{kg}}} \right)} \right)^{{{27}}} \hfill \\ \end{gathered}$$

### The measurement of internal exposure by cesium

The level of ^134^Cs and ^137^Cs was measured as described previously^10^. The level of γ ray in dissected thyroid was measured using a germanium detector (GC4018, CANBELLA, CT, USA). The level of accumulated internal exposure by ^134^Cs or ^137^Cs (U_accum(134 or 137)_) was calculated using the formula below:

$$\begin{gathered} {\text{U}}_{{{\text{sam}}({\text{134 or 137}})}} \left( {\mu {\text{Gy}}/{\text{day}}} \right) \, = {\text{ C}}_{{{\text{sam}}({\text{134 or 137}})}} \left( {{\text{Bq}}/{\text{kg}}} \right) \, \times {\text{ DCF}} \hfill \\ {\text{U}}_{{0({\text{134 or 137}})}} = {\text{ U}}_{{{\text{sam}}({\text{134 or 137}})}} \times {\text{ e}}^{{\lambda {\text{t}}}} \hfill \\ {\text{U}}_{{{\text{accum}}({\text{134 or 137}})}} \left( {{\text{mGy}}} \right) \, = \, \smallint {\text{ U}}_{{({\text{134 or 137}})}} {\text{dt}} \hfill \\ {\text{DCF}}:^{{{134}}} {\text{Cs }} = { 3}.{1 } \times { 1}0^{{ - {3}}} ,^{{{137}}} {\text{Cs }} = { 3}.{7 } \times { 1}0^{{ - {3}}} \left( {\left( {\mu {\text{Gy}}/{\text{day}}} \right)/\left( {{\text{Bq}}/{\text{kg}}} \right)} \right) \hfill \\ \lambda \, \left( {\text{disintegration constant}} \right):^{{{134}}} {\text{Cs }} = { 9}.{19 } \times { 1}0^{{ - {4}}} \left( {/{\text{day}}} \right),^{{{137}}} {\text{Cs }} = { 6}.{31 } \times { 1}0^{{ - {5}}} \left( {/{\text{day}}} \right) \hfill \\ {\text{t}}:{\text{ from the accident to the euthanasia }}\left( {{\text{day}}} \right) \hfill \\ \end{gathered}$$

### Measurement of plasma hormones

The level of plasma hormones was measured using enzyme immunoassays including triiodothyronine (T3; CSB-E13049B, Lot 5312492292, Cusabio, Houston, TX, USA), thyroxine (T4; CSB-E13050B, Lot 256492293, Cusabio), thyroid-stimulating hormone (TSH; CSB-E17548B, Lot Z011229518, Cusabio), cortisol (CSB-E13064B, Lot P31227727, Cusabio), insulin-like growth factor-1 (IGF-1; MBS701164, Lot C1403031439, MyBioSource), and growth hormone (GH; MBS703041, Lot C1403021438, MyBioSource). All the absorbance was measured at 450 nm using a spectrophotometer (ARVO-SV, PerkinElmer Japan, Kanagawa, Japan).

### Real-time PCR analysis

Total RNA in excised thyroid was purified using TRI-Reagent (TR-118, Molecular Research Center, OH, USA) and transferred to cDNA using ReverTra Ace (TRT-101, TOYOBO, Tokyo, Japan). The cDNA was combined with THUNDERBIRD SYBR qPCR Mix (QPS-101, TOYOBO) and amplified with the Aria Mx Real-Time PCR System (Agilent Technologies, Wilmington, DE, USA) under the specific protocol; 1 cycle of 95 °C for 1 min followed by 45 cycles of 95 °C for 15 s and 59 °C for 1 min. The mRNA level of *SLC5A5* was calculated with the ΔΔ Ct method by using *Rn18s* as an internal standard. The primers were shown below; *Rn18s* forward, GACTCAACACGGGAAACCTCAC; *Rn18s* reverse, CACCCACGGAATCGAGAAAG; *SLC5A5* forward, ACCCTCTTCTCACTGCCAAC; *SLC5A5* reverse, GACAAGGGCTCCACATAGCA.

### Statistical analysis

All the experimental results were expressed as mean ± standard error of the mean (SEM). Two-tailed Mann-Whitney U-test was used for two groups, while the Kruskal-Wallis test and the Steel–Dwass' test were used for groups exceeding two groups. For the relationship analysis, we used Spearman's rank correlation test. When the P value was less than 5%, it was determined that there was a significant difference or correlation.

## Supplementary Information

Supplementary Information.

## Data Availability

The map and datasets of ambient dose rate measured with monitoring post are available in the Nuclear Regulation Authority, http://radioactivity.nsr.go.jp/map/ja/. The other datasets generated during and/or analyzed during the current study are available from the corresponding author on reasonable request.
