# Supplementary material for: The effect of exposure on cattle thyroid after the Fukushima Daiichi nuclear power plant accident
Source: Sci Rep. 2022 Dec 16;12:21754. doi: 10.1038/s41598-022-25269-0 (PMC9758204; doi:10.1038/s41598-022-25269-0)
Supplement: Supplementary file 1 — Supplementary Information. [file 41598_2022_25269_MOESM1_ESM.docx]

**The effect of exposure on cattle thyroid after the Fukushima Daiichi nuclear power plant accident**

# Daiki Horikami^1,+^, Naoya Sayama^1,+^, Jun Sasaki^2^, Haruka Kusuno^3^, Hiroyuki Matsuzaki^3^, Akane Hayashi^1^, Tatsuro Nakamura^1^, Hiroshi Satoh^4^, Masahiro Natsuhori^5^, Keiji Okada^6^, Nobuhiko Ito^5^, Itaru Sato^7^, and Takahisa Murata^1,*^

# Supplementary Tables:

| Farm | Cattle | # | Goiter | Atrophy | Dissec time | Age at dissec (year) | Int exp (mGy) | Ext exp (mGy) | Total (mGy) |
| --- | --- | --- | --- | --- | --- | --- | --- | --- | --- |
| Farm A | After 1 | 3 | + | - | May 2013 | Under 2.2 | - | - | - |
|  | After 2 | 5 | - | + | March 2014 | Under 3.0 | - | - | - |
|  | After 3 | 4 | - | - | May 2014 | Under 3.2 | - | - | - |
|  | Before 1 | 1 | - | + | March 2014 | 3.5 | 97 | 995 | 1092 |
|  | Before 2 | 1 | + | - | April 2014 | 3.7 | 97 | 1006 | 1102 |
|  | Before 3 | 2 | + | - | September 2014 | 10.4 ± 0.8 | 99 | 1101 | 1200 |
|  | Before 4 | 3 | - | - | October 2016 | 8.3 ± 1.8 | 103 | 1416 | 1519 |
| Farm B | Before 1 | 2 | - | - | June 2016 | 7.5 ± 0.2 | 35 | - | - |
|  | Before 2 | 1 | - | - | November 2016 | 8.8 | 35 | - | - |
|  | Before 3 | 8 | - | - | April 2017 | 11.1 ± 0.6 | 35 | - | - |
| Hokkaido | Younger | 1 | - | - | May 2017 | 2.5 | - | - | - |
|  | Older | 2 | - | - | May 2017 | 4.2 ± 0.5 | - | - | - |
| Aomori | Younger | 1 | - | - | May 2017 | 2 | - | - | - |
|  | Older | 1 | - | - | May 2017 | 13.9 | - | - | - |
| Iwate | Younger | 60 | - | - | May 2017 | 2.4 ± 0.02 | - | - | - |
|  | Older | 23 | - | - | May 2017 | 13.8 ± 0.8 | - | - | - |
| Miyagi | Younger | 11 | - | - | May 2017 | 2.5 ± 0.02 | - | - | - |
| Fukushima | Younger | 3 | - | - | May 2017 | 2.5 ± 0.01 | - | - | - |

**Table S1. The list of thyroid samples used for this study.**

Dissec, dissection. Int exp, Internal exposure. Ext exp, external exposure.

| Farm | Cattle | # | Goiter | Atrophy | Dissec time | Age at dissec (year) | Int exp (mGy) | Ext exp (mGy) | Total (mGy) |
| --- | --- | --- | --- | --- | --- | --- | --- | --- | --- |
| Farm A | After 1 | 14 | - | - | May 2014 | Under 3.2 | - | - | - |
|  | After 2 | 14 | - | - | September 2014 | Under 3.5 | - | - | - |
|  | After 3 | 19 | - | - | May 2016 | Under 5.2 | - | - | - |
|  | Before 1 | 18 | - | - | May 2014 | 8.2 ± 0.4 | 97 | 1030 | 1127 |
|  | Before 2 | 18 | - | - | September 2014 | 8.4 ± 1.1 | 98 | 1089 | 1187 |
|  | Before 3 | 21 | - | - | May 2016 | 9.1 ± 0.7 | 102 | 1364 | 1466 |
|  | Before 4 | 3 | - | - | October 2016 | 8.1 ± 2.0 | 103 | 1421 | 1524 |
| Farm B | After 1 | 4 | - | - | May 2016 | Under 5.2 | - | - | - |
|  | Before 1 | 7 | - | - | August 2015 | 6.3 ± 1.1 | 35 | - | - |
|  | Before 2 | 10 | - | - | May 2016 | 8.1 ± 1.0 | 35 | - | - |
|  | Before 3 | 7 | - | - | August 2016 | 7.3 ± 1.1 | 35 | - | - |
|  | Before 4 | 7 | - | - | April 2017 | 10.9 ± 0.6 | 35 | - | - |
|  | Before 5 | 7 | - | - | June 2017 | 8.1 ± 1.1 | 35 | - | - |
| Tottori | Younger | 6 | - | - | August 2016 | 4.2 ± 0.2 | - | - | - |
|  | Older | 19 | - | - | August 2016 | 8.9 ± 0.5 | - | - | - |
| Kumamoto | Younger | 9 | - | - | April-June 2017 | 3.5 ± 0.4 | - | - | - |
|  | Older | 10 | - | - | April-June 2017 | 7.8 ± 0.6 | - | - | - |

**Table S2. The list of plasma samples used for this study.**

Dissec, dissection. Int exp, Internal exposure. Ext exp, external exposure.

# Supplementary Figures


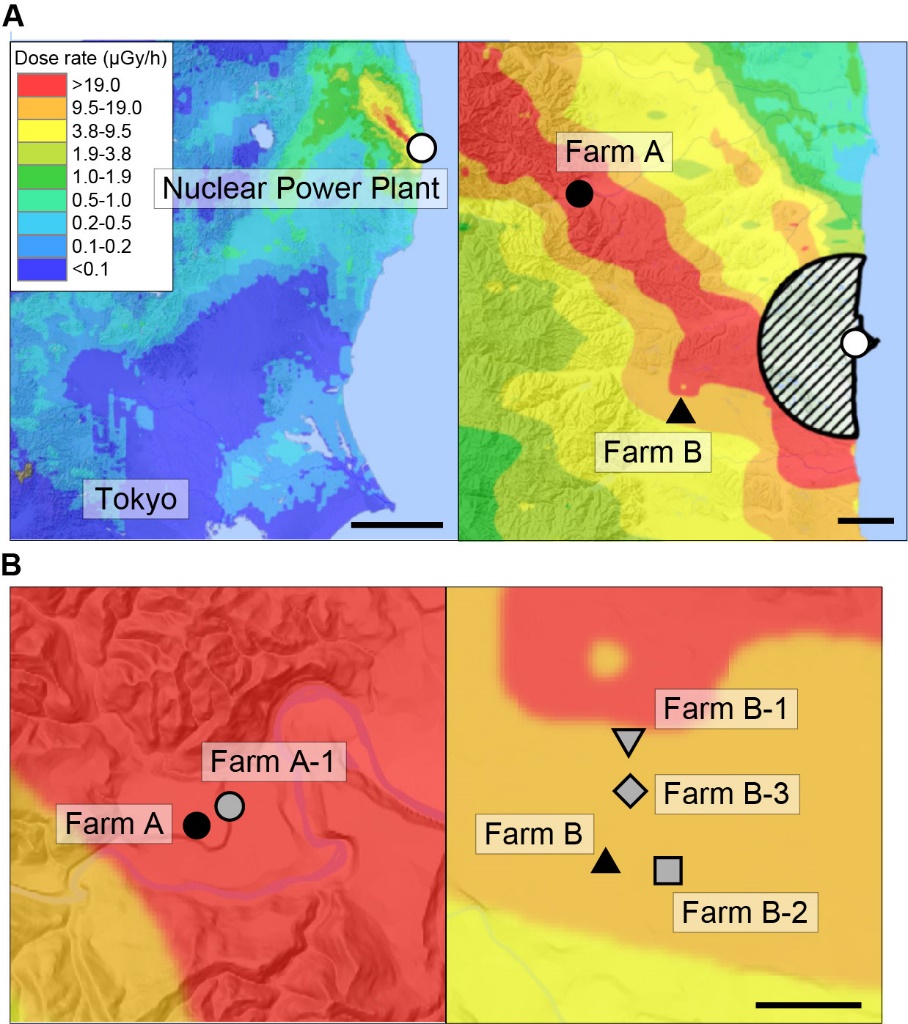


**Figure S1. The place of farm A and farm B.** The place of farm A and farm B in the ‘deliberate evacuation area’. (A) The map in low-power fields (left panel; Scare bar, 50 km) and high-power fields (right panel; Scale bar, 2.0 km). (B) The place of monitoring post around the farms. Scare bar, 500 m. The pictures of the map were created by the processing electronic topographic map 25000 (GSI).


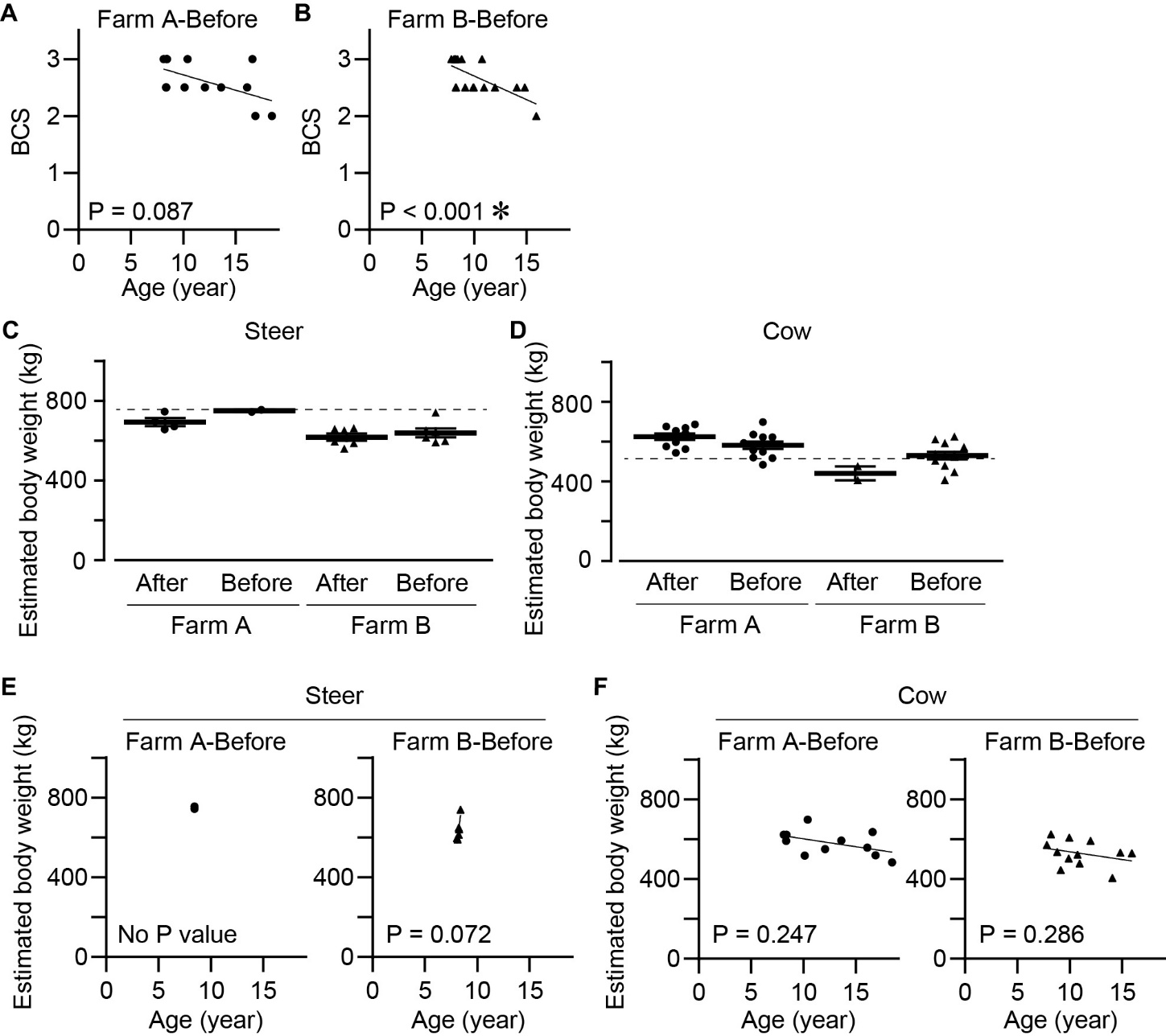


**Figure S2.** **The physique status** **of exposed cattle.** The body conditioning score (BCS) was measured and the body weight of exposed cattle was estimated by measuring body size. (A-B) The scatter plot of age and BCS in (A) Farm A (black circle, n=13) and (B) Farm B (black triangle, n=21). (C-D) The summary of estimated body weight of (C) steer (Farm A, n=4 and 2; Farm B, n=6 each) and (D) cow (Farm A, n=10 and 11; Farm B, n=2 and 12). Broken line, the average weight of Japanese black cattle. (E-F) The scatter plot of age and estimated body weight of (E) steer (Farm A, n=2; Farm B, n=4) and (F) cow (Farm A, n=11; Farm B, n=12). Data in summaries are presented as mean ± SEM. Solid line in scatter plots indicates regression line. *P<0.05.


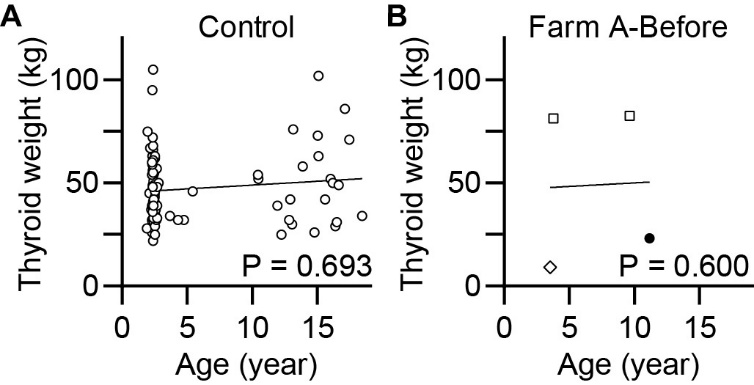


**Figure S3. The thyroid weight of exposed cattle.** Thyroids were dissected and the weight was measured. (A-B) The scatter plot of age and thyroid weight in (A) control farm (open circle, n=102) and (B) Farm A (black circle (normal), open square (goiter), and open rhombus (atrophic thyroid), n=4). Solid line indicates regression line.


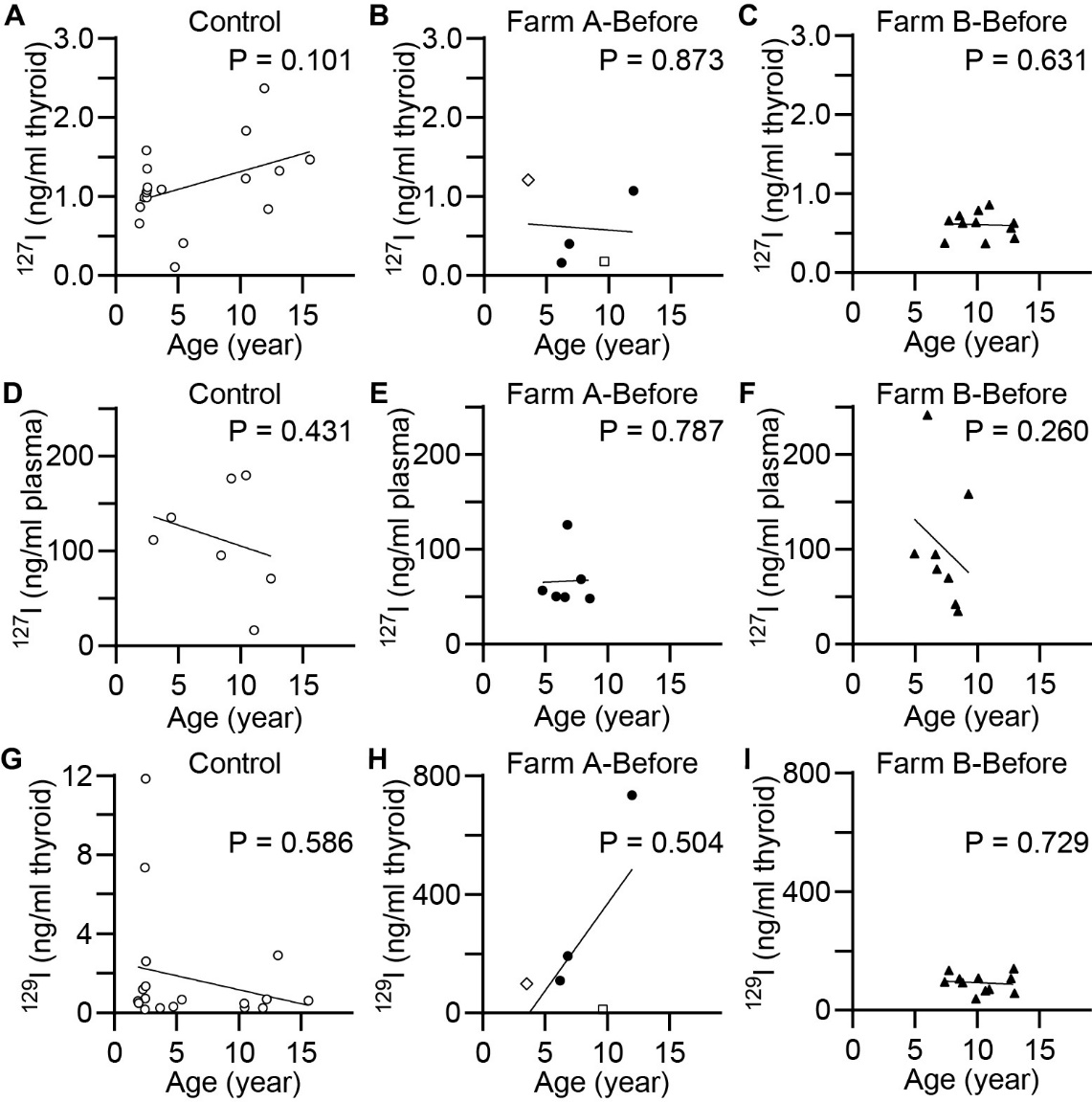


**Figure S4. Internal exposure dose by ^131^I in cattle thyroid.** Thyroids were dissected and the concentrations of ^127^I and ^129^I were measured. (A-C) The scatter plot of age and thyroid ^127^I in (A) control farm (open circle, n=19), (B) Farm A (black circle (normal), open square (goiter), and open rhombus (atrophic thyroid), n=5), and (C) Farm B, black triangle (normal), n=11). (D-F) The scatter plot of age and plasma ^127^I in (D) control farm (n=7), (E) Farm A (n=6), and (F) Farm B (n=8). (G-I) The scatter plot of age and thyroid ^129^I in (G) control farm (n=19), (H) Farm A (n=5), and (I) Farm B (n=11). Solid line indicates regression line.


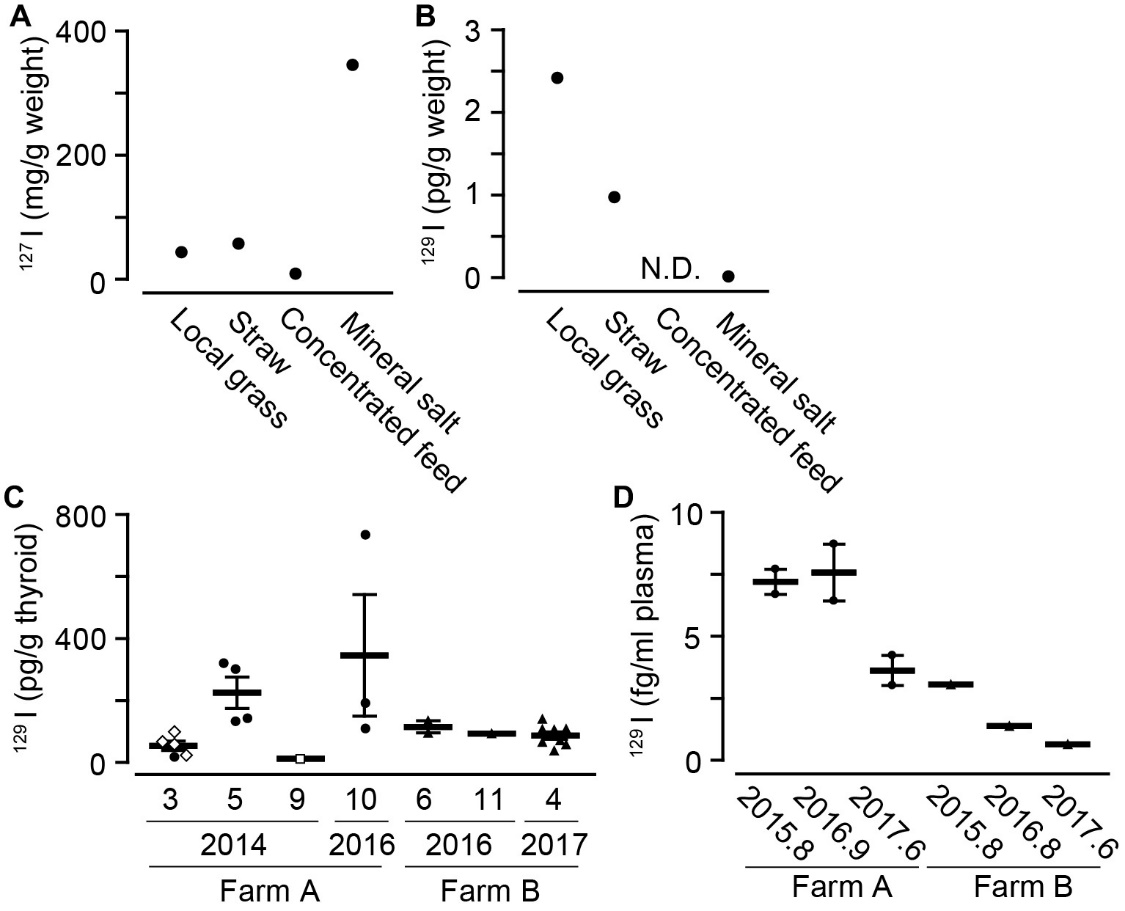


**Figure S5. The level of ^127^I and ^129^I in food, thyroid, and plasma.** (A and B) Local grass, straw, concentrated feed, and mineral salt in farm A were taken and the level of (A) ^127^I and (B) ^129^I was measured (n=1 each). (C) The summary of thyroid ^129^I divided in sampling time (Farm A, black circle, open square (goiter), and open rhombus (atrophic thyroid), n=6, 4, 1, and 3; farm B, black triangle, n=2, 1, and 8). (D) The summary of plasma ^129^I (Farm A, black circle, n=2 each; farm B, black triangle, n=1 each). Data are presented as mean ± SEM.


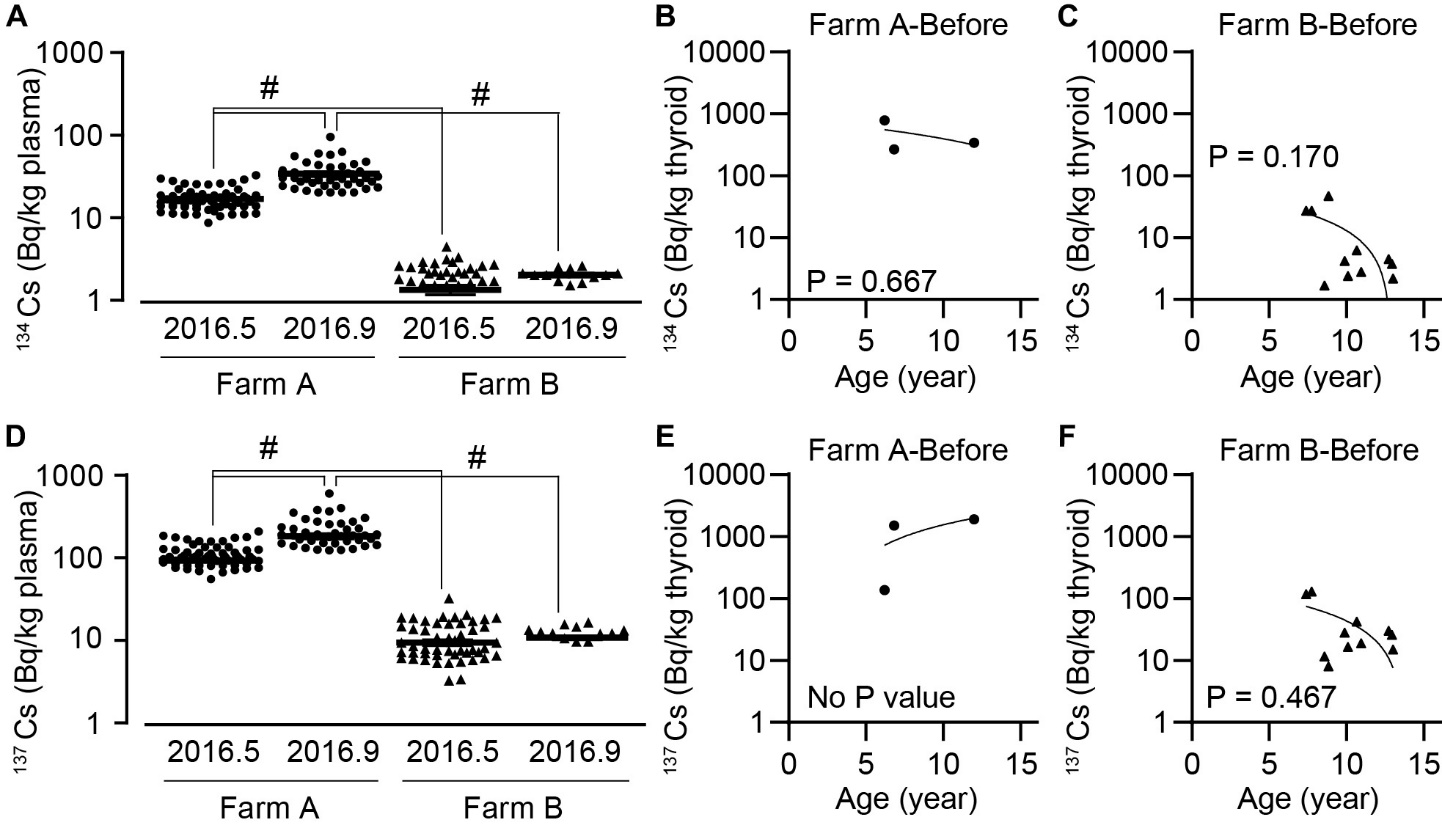


**Figure S6. Internal exposure dose by ^134^Cs and ^137^Cs in cattle plasma and thyroid.** Thyroids were dissected and the levels of ^134^Cs and ^137^Cs were measured. (A) The summary of plasma ^134^Cs (Farm A, black circle, n=61 and 43; farm B, black triangle, n=49 and 12, respectively). (B-C) The scatter plot of age and becquerel of thyroid ^134^Cs in (B) Farm A (n=3) and (C) Farm B (n=11). (D) The summary of plasma ^137^Cs (Farm A, n=61 and 43; farm B, n=49 and 12). (E-F) The scatter plot of age and becquerel of thyroid ^137^Cs in (E) Farm A (n=3) and (F) Farm B (n=11). Data in summaries are presented as mean ± SEM. #P<0.05. Solid line in scatter plots indicates regression line.


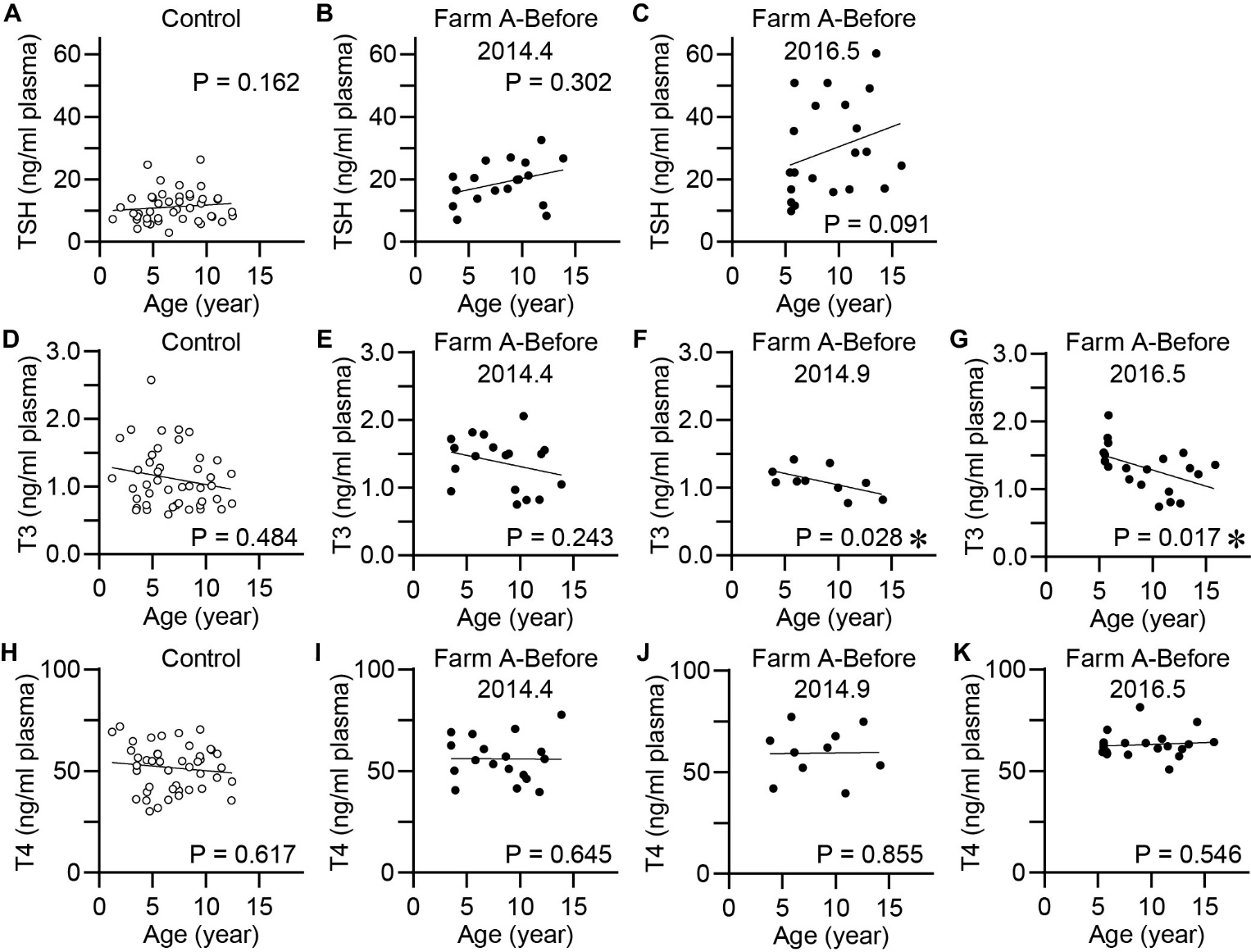


**Figure S7. The level of thyroid hormones in plasma.** Plasma was collected in 2014-2016 and the levels of thyroid related hormones were measured. (A-C) The scatter plot of age and TSH in (A) control farm (open circle, n=44), (B) Farm A taken in April 2014, black circle, n=18), and (C) in May 2016 (n=21). (D-G) The scatter plot of age and T3 in (D) control farm (n=44), (E) Farm A in April 2014 (n=18), (F) in September 2014 (n=10), and (G) in May 2016 (n=21). (H-K) The scatter plot of age and T4 in (H) control farm (n=44), (I) Farm A in April 2014 (n=18), (F) in September 2014 (n=10), and (G) in May 2016 (n=21). Solid line indicates regression line. *P<0.05.

**
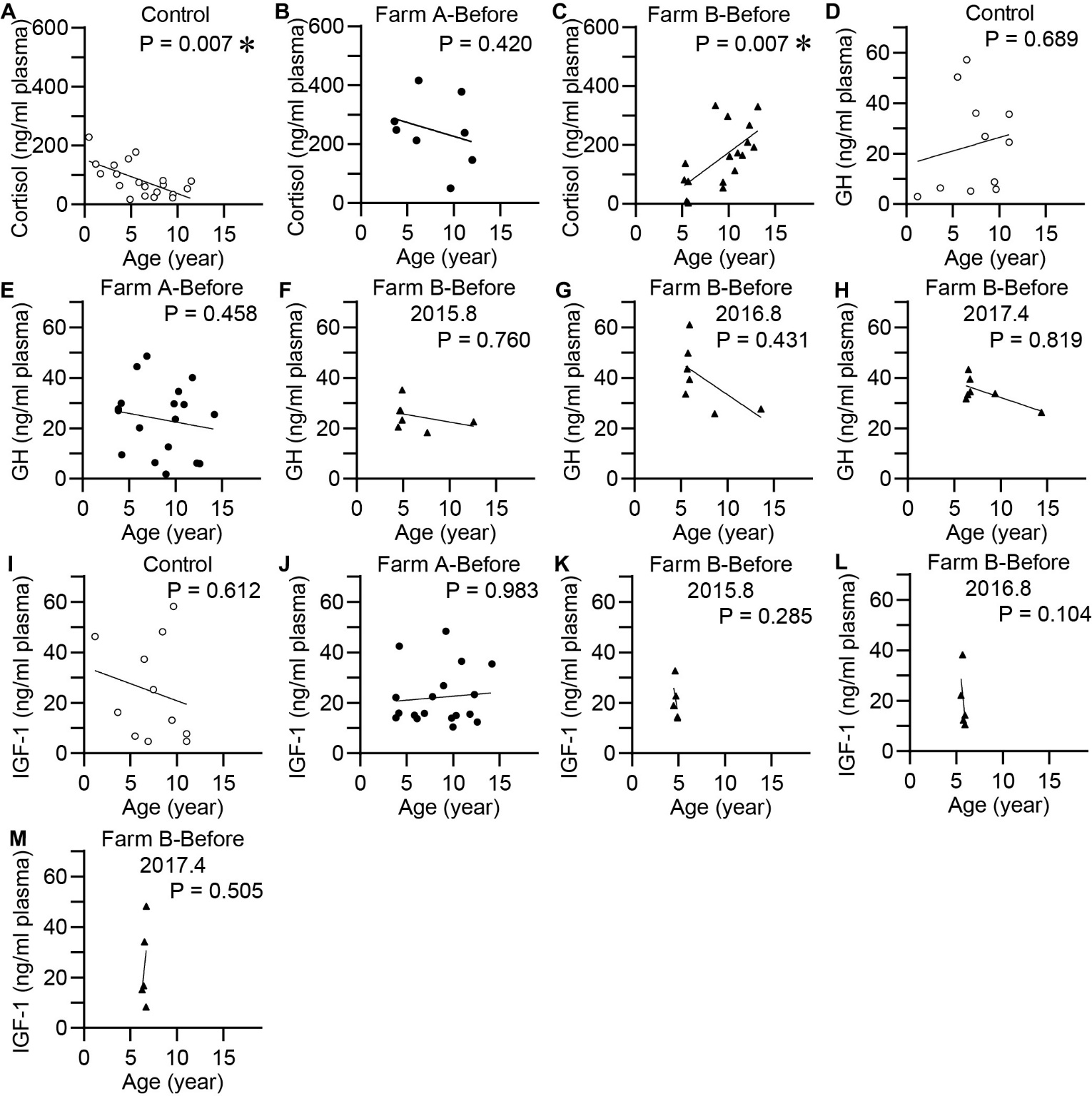
**

**Figure S8. The concentration of anterior pituitary-derived hormones in plasma.** Plasma was collected and the levels of anterior pituitary-derived hormones were measured. (A-C) The scatter plot of age and plasma cortisol in (A) control farm (open circle, n=21), (B) Farm A (black circle, n=9), and (C) Farm B (black triangle, n=17). (D-H) The scatter plot of age and plasma GH in (D) control farm (n=11), (E) Farm A (n=18), and (F-H) Farm B (n=7 each). (I-M) The scatter plot of age and plasma IGF-1 in (I) control farm (n=11), (J) Farm A (n=18), and (K-M) Farm B (n=5 each). Solid line indicates regression line. *P<0.05.


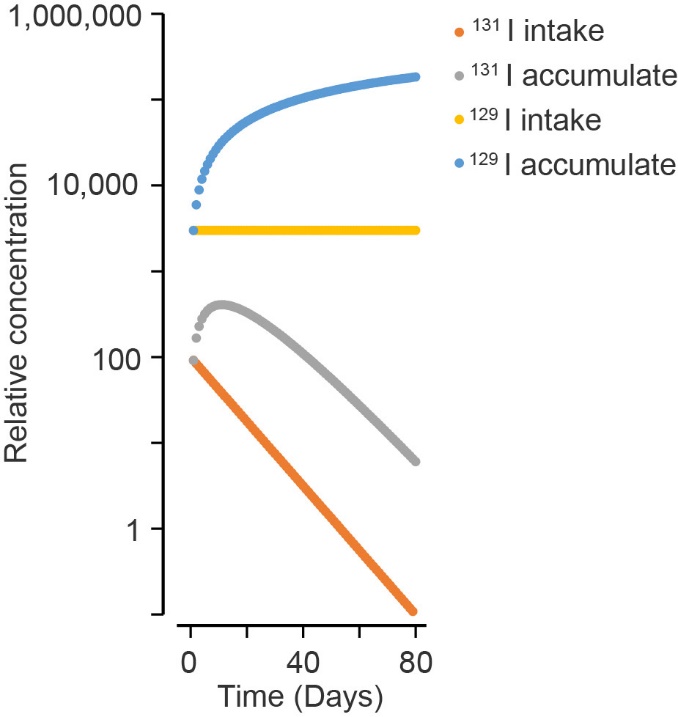


**Figure S9. The model of the concentration of ^129^I and ^131^I in thyroid after the FDNPP accident.** After the FDNPP accident, Radioactive iodine were released (^129^I:^131^I =30:1). Released ^129^I and ^131^I were taken and accumulated into cattle thyroid. The level of ^131^I drops by the collapse, while the level of ^131^I reaches to equilibrium.
